# Reflection Absorption Infrared Spectroscopy Characterization of SAM Formation from 8-Mercapto-*N*-(phenethyl)octanamide Thiols with Phe Ring and Amide Groups

**DOI:** 10.3390/molecules25235633

**Published:** 2020-11-30

**Authors:** Zenonas Kuodis, Ieva Matulaitienė, Marija Špandyreva, Linas Labanauskas, Sigitas Stončius, Olegas Eicher-Lorka, Rita Sadzevičienė, Gediminas Niaura

**Affiliations:** Department of Organic Chemistry, Center for Physical Sciences and Technology (FTMC), Saulėtekis Ave. 3, LT-10257 Vilnius, Lithuania; zenonas.kuodis@ftmc.lt (Z.K.); ieva.matulaitiene@ftmc.lt (I.M.); marija.spandyreva@gmail.com (M.Š.); linas.labanauskas@ftmc.lt (L.L.); sigitas.stoncius@ftmc.lt (S.S.); olegas.eicher-lorka@ftmc.lt (O.E.-L.); rita.sadzeviciene@ftmc.lt (R.S.)

**Keywords:** RAIRS, SAM, phenylalanine ring, gold, amide

## Abstract

Multifunctional amide-containing self-assembled monolayers (SAMs) provide prospects for the construction of interfaces with required physicochemical properties and distinctive stability. In this study, we report the synthesis of amide-containing thiols with terminal phenylalanine (Phe) ring functionality (HS(CH_2_)_7_CONH(CH_2_)_2_C_6_H_5_) and the characterization of the formation of SAMs from these thiols on gold by reflection absorption infrared spectroscopy (RAIRS). For reliable assignments of vibrational bands, ring deuterated analogs were synthesized and studied as well. Adsorption time induced changes in Amide-II band frequency and relative intensity of Amide-II/Amide-I bands revealed two-state sigmoidal form dependence with a transition inflection points at 2.2 ± 0.5 and 4.7 ± 0.5 min, respectively. The transition from initial (disordered) to final (hydrogen-bonded, ordered) structure resulted in increased Amide-II frequency from 1548 to 1557 cm^−1^, which is diagnostic for a strongly hydrogen-bonded amide network in trans conformation. However, the lateral interactions between the alkyl chains were found to be somewhat reduced when compared with well-ordered alkane thiol monolayers.

## 1. Introduction

Self-assembled monolayers (SAMs) of functional thiol molecules at the gold metal surface modify interfacial physicochemical properties and provide a valuable platform for the investigation of specific interactions of the terminal functional group with solution components, provide the possibility to probe the mechanism of electron transfer reactions, and serve for the construction of (bio)sensors and the development of biotechnological processes [1,2,3,4]. The terminal group of monolayers affords the opportunity to construct an interface with specifically designed physical and chemical properties. The monolayer containing the terminal phenylalanine (Phe) ring group provides the possibility to probe the subtle interactions of the ring with solution components and adjacent adsorbed molecules in the monolayer. The Phe ring plays an important role in stabilizing the tertiary structure of proteins, binding, and the catalytic function [5]. Being fully aromatic, Phe residue participates in a variety of noncovalent interactions involving the delocalized π-electron system, such as π-π stacking, CH-π, and cation-π [6,7,8,9]. These interactions are weak and difficult to study. Self-assembled monolayers on the gold surface with the terminal Phe ring group may provide a promising platform to gain insights into the various interactions of aromatic moiety that are important for the biological function of proteins and peptides.

The functional properties of the terminal molecular group depend on the structure and stability of the monolayer. The architecture and stability of SAM depends on several important interactions, the main ones being (i) metal-sulfur covalent bonding, (ii) interactions between the adjacent hydrocarbon chains, and (iii) interactions between the terminal functional groups in the monolayer and with solution components. The stability of the monolayer increases considerably by the introduction of the amide functional group in the hydrocarbon chain of thiol molecules because of the formation of lateral hydrogen bonds between the adjacent chains in the monolayer [10,11,12,13,14,15,16]. Indeed, temperature-programmed desorption studies have revealed that the amide group containing SAM displayed a delay of the alkyl chain disordering by 50 K, as compared to the linear chain thiols with OH-terminal group. In addition, the onset of sulfur desorption has been found to occur at 25 K higher temperatures [15]. It was demonstrated that the presence of the hydrogen bonding network increases the ordering of alkanethiols and the blocking properties of the monolayer for electrochemical reactions [13].

Several analytical techniques have been used extensively to characterize self-assembled monolayers, such as high-resolution electron energy loss spectroscopy (HREELS), X-ray photoelectron spectroscopy (XPS), reflection absorption infrared spectroscopy (RAIRS), sum-frequency generation (SFG) spectroscopy, surface-enhanced Raman spectroscopy (SERS), atomic force microscopy (AFM), scanning tunneling microscopy (STM), and cyclic voltammetry [1,2,17]. Among these techniques, the RAIRS approach is particularly useful for acquiring vibrational information from monolayers on smooth gold substrates because of the submonolayer sensitivity and ability to attain molecular level information on the hydrogen bonding interaction and ordering of molecules in the film [18,19,20,21,22,23]. In fact, monitoring the stretching frequency of methylene bands is one of the most sensitive methods for the analysis of the conformational order of alkyl chains [24,25]. An important advantage of the RAIRS tool for analysis of monolayers on a metallic surface is the operation of specific surface selection rules, stating that only the vibrations with a transition dipole moment component aligned perpendicularly to the surface are active for adsorbed molecules [26]. Therefore, the method affords important information about the orientation of molecules and molecular groups with respect to the surface and bonding geometry [27,28,29].

**Figure 1:**
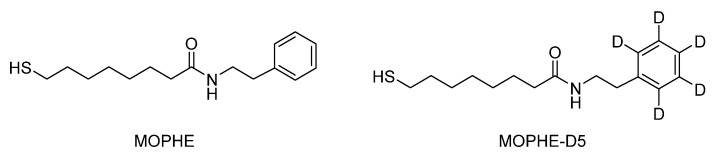
Molecular structures of multifunctional thiol compounds MOPHE (8-mercapto-***N***-(phenethyl)octanamide) and MOPHE-D_5_ (8-mercapto-*N*-(phenethyl)octanamide-*d*_5_).

The present work aims at the reflection absorption infrared spectroscopy characterization of monolayers formed from newly synthesized multifunctional thiol molecules with the Phe ring terminal group and intrachain amide moiety (Figure 1).

## 2. Results and Discussion

### 2.1. Assignments of MOPHE Infrared Absorption bands

**Figure 2:**
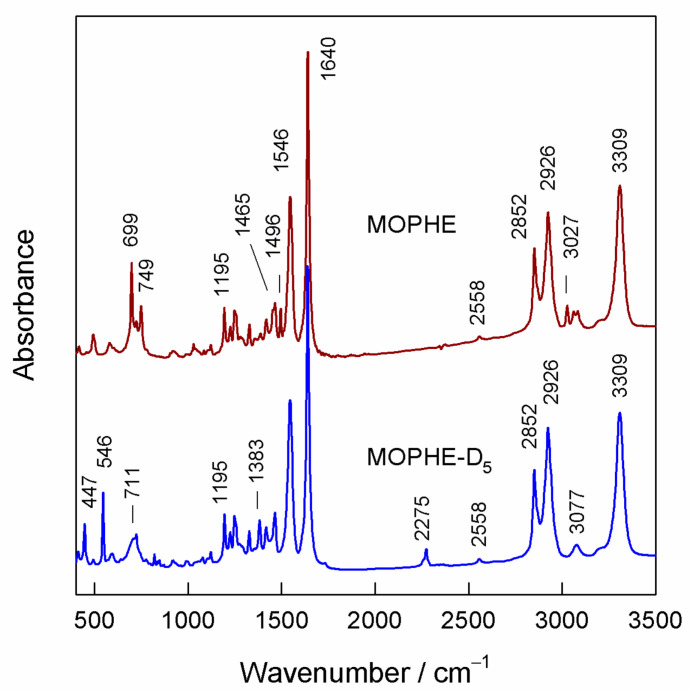
Infrared spectra of bulk MOPHE and Phe ring deuterated MOPHE-D_5_ compounds.

The MOPHE molecule consists of four molecular units: (i) the thiol group (SH), (ii) the polyethylene chain (−(CH_2_)_7_−), (iii) the amide group (−CO−NH−), and (iv) the terminal Phe ring (Figure 1). Each molecular group can be characterized by infrared spectroscopy. To increase reliability and facilitate the assignments of infrared absorption bands, we have synthesized the studied thiol molecule with deuterated terminal Phe ring, MOPHE-D_5_. Thus, ring-related vibrational modes can be easily identified by an observed frequency shift. To clarify the assignments of infrared bands, we have conducted the first-principles quantum chemical calculations of MOPHE and MOPHE-D_5_ compounds. Figure 2 compares infrared absorption spectra of bulk MOPHE and MOPHE-D_5_ compounds dispersed in the KBr tablet. Assignments of the main infrared bands based on our calculations and previous publications [30,31,32,33,34,35,36,37,38,39] are given in Table 1.

The most intense bands are associated with the vibrations of the amide group. The highest frequency intense band near 3309 cm^−1^ belongs to stretching vibration ν(N−H) (Amide-A mode, Am-A). The frequency of this mode decreases with the increasing hydrogen bonding interaction strength with the hydrogen atom of amide group. The most intense band located at 1640 cm^−1^ is primarily associated to C=O stretching vibration (83%) coupled with out-of-phase C−N stretching and the C–C−N deformation motion of the amide group (Amide-I mode, Am-I) [31,32,33,34]. The well-defined band at 1546 cm^−1^ is associated with the coupled out-of-phase C−N stretching and in-plane N−H bending vibrations (Amide-II mode, Am-II) [34]. The Amide-III (Am-III) vibrational mode is visible as the middle intensity band near 1249 cm^−1^. This mode originates primarily from the in-phase C−N stretching vibration coupled with the N−H in-plane bending. Finally, the low frequency Amide-V vibrational mode (Am-V) associated with the N−H out-of-plane bending vibration is visible near 711 cm^−1^. Because of the high number of methylene groups, several modes associated with the alkyl chain vibrations are clearly visible in the infrared spectra. Thus, intense bands at 2852 and 2926 cm^−1^ are associated with symmetric stretching and asymmetric stretching vibrations of the CH_2_ groups, respectively (Figure 2, Table 1). The deformation (scissoring) vibration of the CH_2_ groups is clearly visible as a middle intensity band near 1465 cm^−1^. Other relatively low intensity deformation vibrations of methylene groups are listed in Table 1. The thiol group can be recognized in the infrared spectra from the low intensity S−H stretching band near 2558 cm^−1^. For thiol compounds dissolved in water, this band usually appears at 2582 cm^−1^ [4]; a shift to lower wavenumbers indicates a strong hydrogen bonding interaction of the thiol groups in the solid state. Because of the relatively low intensity, the Phe ring vibrational modes can only be recognized in the infrared spectra by ring-deuteration-induced frequency shifts. Thus, aromatic ring stretching vibration ν(=C–H) shifts from 3027 to 2275 cm^−1^ upon Phe ring-deuteration. The characteristic substituted benzene ring C=C stretching mode coupled with in-plane C–H deformation, named F19 (according to Wilson notation for Phe ring) appears at 1496 and 1383 cm^−1^ in the spectra of the MOPHE and MOPHE-D_5_ compounds, respectively. The middle intensity out-of-plane C–H deformation vibrational modes F11 and F4 of the MOPHE ring are visible at 749 and 699 cm^−1^, respectively.

### 2.2. Reflection Absorption Infrared Spectroscopy (RAIRS) Analysis of Monolayer Formation

**Figure 3:**
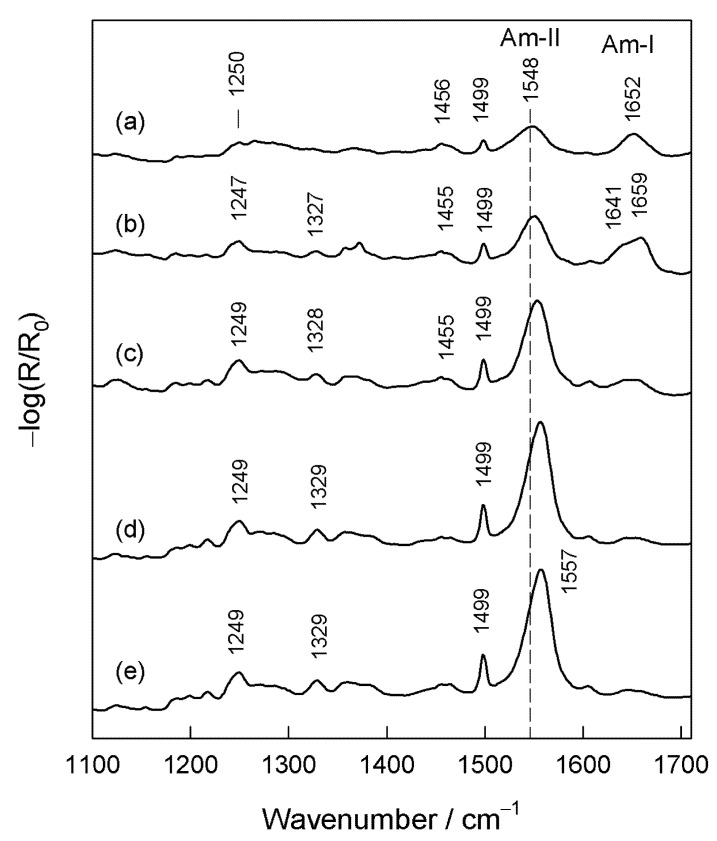
Reflection absorption infrared spectroscopy (RAIRS) spectra of MOPHE adsorbed at the gold surface in the fingerprint spectral region (1100–1710 cm^−1^) obtained at different immersion times in ethanol solution containing 10^−3^ M MOPHE. Immersion time: (**a**) 5 s, (**b**) 30 s, (**c**) 3 min, (**d**) 1 h, and (**e**) 24 h.

Figure 3 shows the immersion-time in adsorption solution-dependent RAIRS spectra of the adsorbed MOPHE compound on the gold surface. After 5 s incubation in the adsorption solution, the RAIRS spectrum reveals two broad and similar intensity bands at 1652 and 1548 cm^−1^ which are associated with the Am-I and Am-II vibrational modes of the amide group, respectively (Figure 3a). The presence of the Phe ring at the interface can be recognized from the narrow and low intensity F19 mode at 1499 cm^−1^. The band near 1456 cm^−1^ belongs to the scissoring deformation vibration of CH_2_ groups in the hydrocarbon chain. The frequency of the Am-I mode is a sensitive indicator of the hydrogen bonding interaction (C=O∙∙∙H) strength involving the amide group and dipole–dipole interaction between the C=O groups [32,33,38]. Higher Am-I frequency corresponds to weakened hydrogen bonding interaction [38]. A relatively low Am-I wavenumber value for the MOPHE compound in the solid state (1640 cm^−1^) resembles the strongly hydrogen-bonded network of molecules. However, at the initial monolayer formation stage, the peak position of this band is upshifted (1652 cm^−1^), indicating a weakened hydrogen bonding interaction strength through the C=O group of the amide moiety. It has been demonstrated previously that, contrary to Am-I, the frequency of the Am-II mode probes the hydrogen bonding interaction strength primarily at the N−H site of the amide group [40]. The formation of stronger hydrogen bonds at this site results in higher Am-II frequencies. At the initial adsorption state (Figure 3a), the wavenumber value of Am-II band (1548 cm^−1^) is very similar with the one observed for MOPHE in the solid state (1546 cm^−1^); thus at this condition, the N−H site is involved in a strong interaction. Such an interaction may not be related solely with the hydrogen bonding interaction, but in addition, may include contact with the metal or even bonding with the gold surface through the amide moiety [41,42].

The increase of the incubation time in the adsorption solution to 30 s results in the intensity increase of all the vibrational bands (Figure 3b). In addition, the broad band near 1247 cm^−1^ becomes clearly visible. This band is associated with the Am-III vibrational mode of the amide group [43,44]. The frequency of this band is sensitive to the hydrogen bonding interaction at both the C=O and N−H sites of amide group. While the intensity of this band usually is lower by 5–10 times compared with the Am-I mode in the infrared spectra of the polypeptides and proteins, this mode provides useful information on the secondary structure of biomolecules because of the absence of interference with the water contribution and better resolved positions of secondary structure elements [33,45]. It should be noted that in the RAIRS spectrum of MOPHE obtained after 30 s incubation time, the Am-I band splits into the two components positioned at 1641 and 1659 cm^−1^, indicating the presence of two differently hydrogen-bonded populations of adsorbed molecules on the gold surface. The lower frequency component resembles strongly hydrogen-bonded amide groups through the C=O moiety. The further increase of the incubation time of gold substrate in the adsorption solution results in considerable changes in relative intensities and other parameters of the Am-I and Am-II bands.

**Figure 4:**
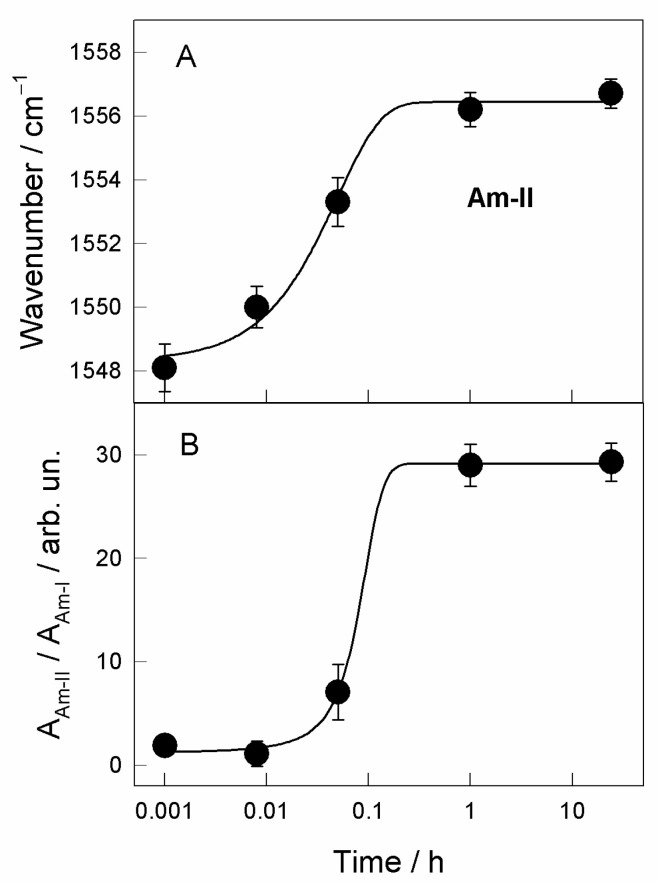
Dependence of parameters of RAIRS bands: (**A**) Amide-II (Am-II) mode wavenumber and (**B**) relative integrated intensity *A*_Am-II_/*A*_Am-I_ ratio on immersion time in ethanol solution containing 10^−3^ M of MOPHE compound. The solid lines are the best fit to Equation (1) with transition inflection point values *t*_m_ = 2.2 ± 0.5 min and *t*_m_ = 4.7 ± 0.5 min for graphs (**A**) and (**B**), respectively.

**Figure 5:**
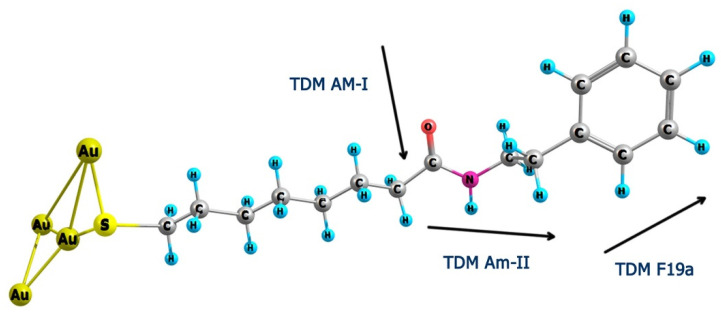
Structure of Au_4_−MOPHE complex optimized at DFT/B3LYP/6-311++G(2d,p) basis set for C, H, N, O, and S atoms and LANL2DZ with ECP for Au atoms and transition dipole moment (TDM) directions for Am-I, Am-II, and Phe ring F19a vibrational modes.

A more detailed analysis of the incubation time-induced changes in the parameters of amide bands is displayed in Figure 4. One can see that both the peak position of the Am-II band and ratio of integrated intensities *A*_Am-II_/*A*_Am-I_ dependencies on the incubation time in the adsorption solution exhibit a similar sigmoidal form. Thus, experimental data were fitted with the equation representing a two-state mechanism for the changes in the frequency and relative intensity of Am-II band with four parameters [38]:

(1) $$P=P_{0}+\frac{\alpha }{\left(1+exp\left(-\frac{t-t_{m}}{b}\right)\right)}$$

where *t*_m_ is the observed transition inflection point. The best fit to Equation (1) was found to be with *t*_m_ = 2.2 ± 0.5 min and *t*_m_ = 4.7 ± 0.5 min for the Am-II frequency and relative intensity *A*_Am-II_/*A*_Am-I_ dependencies on incubation time in adsorption solution, respectively. Presented spectroscopic data show that the Am-II frequency increases with the development of SAM at the gold surface. A higher frequency indicates a stronger hydrogen bonding interaction strength at the N−H site of the amide group [40]. Clegg et al. [13] have proposed that the upshift in frequency of the Am-II mode specifies the increasing restriction forced on the N−H bending vibration within the SAM, consisting of the presence of the strong hydrogen bonding network. The high peak frequency of the Am-II band observed after long adsorption time (1557 cm^−1^) (Figure 4A) is consistent with the presence of strongly hydrogen-bonded amide groups in trans configuration [44,46]. Thus, two states of the amide group N−H site can be represented as the initial relatively weak and final relatively strong hydrogen bonding interactions. The intensities of the Am-I and Am-II modes in the RAIRS method depend on the orientation of the amide group with respect to the surface plane. In general, the relative intensity of the band in the RAIRS spectrum is proportional to the transition dipole moment (TDM) component perpendicular to the surface plane [21,22]. The transition dipole moment component for the Am-I mode lies perpendicularly to the long molecular axis; in contrast, parallel orientation is characteristic for the Am-II mode [31,43]. DFT calculations of the model complex compound Au_4_−MOPHE show the TDM of the Am-I mode aligned nearly perpendicularly to the main molecular axis of –(CH_2_)_7_− hydrocarbon chain, while the TDM of the Am-II mode was found to be ranged in near parallel with this axis configuration (Figure 5). Therefore, the variation in orientation of the molecular axis of the adsorbed thiol molecules containing the amide group from near parallel with respect to the surface plane to a near perpendicular alignment results in the increase of Am-II and the decrease of Am-I band intensities [47]. Relative intensity changes depicted in Figure 4B evidence a sharp transition in the orientation of adsorbed molecules from the predominant near parallel orientation of the molecular axis at short incubation times to a near perpendicular configuration after a longer adsorption period. The reorientation transition inflection time was found to be 4.7 ± 0.5 min. One can see that the intensity of the Am-I band is very low for the RAIRS spectra recorded at long adsorption time (Figure 3). In accordance with the surface selection rules for RAIRS spectroscopy, this observation confirms a near parallel with surface plane orientation of the C=O group and a close to surface normal orientation of the long molecular axis of the studied thiol compound (Figure 5) [21,22,43].

In addition, we observed adsorption time-induced changes in the width of the bands determined as full width at half maximum (FWHM). Thus, the FWHM value of the Am-II band decreases considerably from 37.5 cm^−1^ to 28.8 cm^−1^ as the adsorption time increases from 5 s to 1 h, respectively. The narrowing of the band suggests a homogeneous and well-packed structure without a high number of gauche defects in the main molecular chain. The Am-II band shape is symmetric (Figure 3); and there is no lower frequency band near 1510 cm^−1^, due to the unbounded or weakly-bonded amide groups being visible [10,46].

Insights into the alignment of the terminal Phe ring may be gained from the analysis of the prominent band at 1499 cm^−1^ (F19a). Figure 3 shows that the relative intensity of this band clearly increases with increasing the incubation time. The TDM of this mode is aligned primarily along with the C–C_6_H_5_ bond (Figure 5). In the spectrum of the bulk MOPHE compound intensity of F19a mode is considerably lower compared to the Am-I mode (Figure 2). However, the surface spectrum intensity of this mode is substantially higher compared to Am-I (Figure 3). Thus, changes in the relative intensity of F19a band during the self-assembly imply a near perpendicular with surface orientation of C–C_6_H_5_ bond at the final monolayer formation stage.

**Figure 6:**
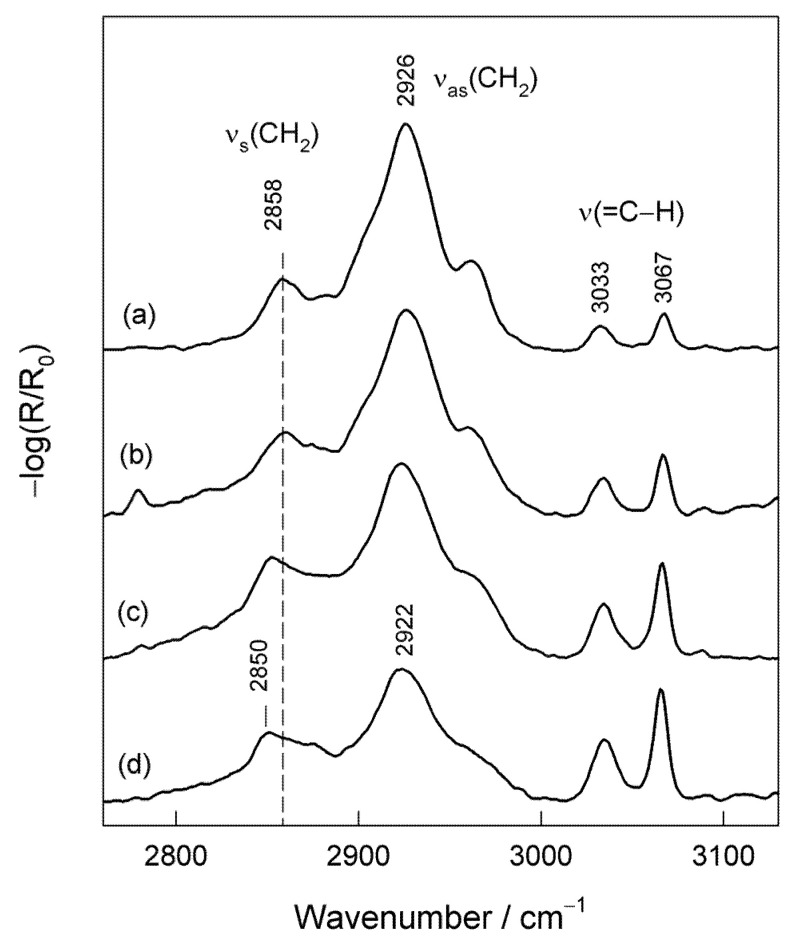
RAIRS spectra of MOPHE adsorbed at the gold surface in the C–H stretching spectral region (2750−3150 cm^−1^) obtained at different immersion times in ethanol solution containing 10^−3^ M MOPHE. Immersion time: (**a**) 5 s, (**b**) 30 s, (**c**) 3 min, and (**d**) 1 h.

Information on the structure and packing of the alkyl chain in the monolayer might be extracted from the analysis of the high frequency C–H stretching vibration region of the RAIRS spectra (Figure 6). At initial adsorption time (5 s), the intense band near 2926 cm^−1^, due to asymmetric stretching vibration of CH_2_ groups (d^−^), dominates in the spectrum. The symmetric stretching vibration of methylene groups is visible near 2858 cm^−1^ (d^+^), while two bands at 3033 and 3067 cm^−1^ are associated with stretching vibration ν(=C–H) and demonstrate the presence of the terminal Phe ring at the interface [39]. The high frequency value of ν_as_(CH_2_) and ν_s_(CH_2_) modes indicates the disordered state of the alkyl chains at the interface [12,15,48]. It is known that for SAMs with well-ordered and densely packed alkyl chains, the diagnostic positions of methylene stretching vibrations ν_as_(CH_2_) and ν_s_(CH_2_) occur at lower wavenumbers, i.e., at 2918 and 2950 cm^−1^, respectively [12]. A relatively high intensity of the ν_as_(CH_2_) band is consistent with the alignment of alkyl chains at a high angle to the normal surface, because the transition dipole moment of this mode is perpendicular to C–C bond. The increase in adsorption time results in the substantial reorganization of alkyl chains in the monolayer: (i) relative intensity of ν_as_(CH_2_) mode decreases, (ii) frequency of ν_as_(CH_2_) and ν_s_(CH_2_) bands shifts significantly to lower wavenumbers, and (iii) relative intensity of terminal Phe ring modes at 3033 and 3067 cm^−1^ increases (Figure 6). Observed changes indicate the development of ordering and packing in the alkyl chain region of the monolayer. However, it should be noted that even after 24 h adsorption time, the frequency of ν_as_(CH_2_) mode did not reach the diagnostic value for well-ordered and highly-packed structures, 2918 cm^−1^ [12]. Snyder et al. [24,49] and Troughton et al. [50] have demonstrated that the position of ν_as_(CH_2_) frequency provides insight into the extent of the lateral intermolecular interactions between the alkyl chains. The increase in lateral interactions downshifts the frequency. Thus, lateral interactions between the alkyl chains are somewhat reduced in the case of our studied monolayer when compared with the densely packed long-alkyl chain structures, possibly because of the bulky terminal Phe ring group.

## 3. Conclusions

We synthesized a novel multifunctional thiol compound with terminal phenylalanine and intrachain amide groups (HS(CH_2_)_7_CONH(CH_2_)_2_C_6_H_5_). To provide reliable assignments of the bands, the isotopically substituted analog with the deuterated phenylalanine ring was synthesized as well. The adsorption time-dependent evolution of the structure of the self-assembled monolayer was probed by reflection absorption infrared spectroscopy. We have observed a sigmoidal form transition from unordered to strongly hydrogen-bonded amide functionality with a transition inflection point at 2.2 ± 0.5 min related with the Amide-II band frequency increase from 1548 to 1557 cm^−1^. In addition to the Amide-II frequency evolution, we observed the sigmoidal form increase in the relative integrated intensity of the Amide-II/Amide-I bands, suggesting the adsorption time-induced reorientation of molecules in the monolayer so that, in the final state, the amide group C=O and N−H bonds are positioned nearly parallel to the gold substrate plane. No Amide-II band characteristic for unbounded or weakly bonded amide groups (near 1510 cm^−1^) was detected in the RAIRS spectra after sufficiently long adsorption times. The frequency of asymmetric stretching vibration of CH_2_ groups (2922 cm^−1^) revealed that the alkyl chain region is not as compact as was previously observed for long-chain well-ordered alkanethiol monolayers. On the basis of the changes in the relative intensity of F19a band of Phe ring at 1499 cm^−1^ during the self-assembly it was suggested that the C–C_6_H_5_ bond adopts a near perpendicular with surface plane orientation at the final monolayer formation stage.

We intend to extend the vibrational spectroscopy study of SAMs formed from MOPHE and MOPHE-D_5_ compounds in spectroelectrochemical cell by using surface-enhanced Raman spectroscopy (SERS) [51] to gain insights into the electric potential-induced changes in the structure of the Phe ring and amide groups.

## 4. Materials and Methods

### 4.1. Materials

Millipore-purified water (18 MΩ cm) was used for the preparation of monolayer formation solutions. The used inorganic salts were of ACS grade and were purchased from Sigma-Aldrich Chemie GmbH (Schnelldorf, Germany). Gold substrates were prepared by magnetron sputtering of the Cr sublayer (~3 nm) and, subsequently, on the 200 nm layer of Au on the cleaned glass substrates. These substrates were immersed into a 10 mM MOPHE ethanol solution for various adsorption times. Subsequently, the substrates were rinsed with ethanol (≥99.8%) and dried under N_2_ flow.

### 4.2. Reflection-Absorption IR Spectroscopy (RAIRS)

RAIRS data were collected on a Vertex 80v FTIR spectrometer (Bruker Inc., Ettlingen, Germany) equipped with a liquid nitrogen cooled mercury-cadmium-telluride (MCT) narrow band detector and a horizontal reflection accessory [52]. The spectral resolution was set at 4 cm^−1^. Spectra were acquired from 400 scans at a grazing angle of 80° by using p-polarized light. The spectrometer and the sample chamber were evacuated during the measurements. The spectrum of hexadecanethiol-d_33_ [HS(CD_2_)_15_CD_3_] self-assembled on gold was used as a reference. Infrared measurements of bulk samples were recorded by using KBr pellets. Frequencies and intensities of RAIRS bands were determined by fitting the experimental contour with Gaussian-Lorentzian form components. Spectral analysis was performed by using GRAMS/A1 8.0 (Thermo Electron Corp., Waltham, MA, USA) software. Standard deviations of parameters of RAIRS bands (peak position and intensity) were determined from 3–5 measurements.

### 4.3. Theoretical Modeling

Theoretical modeling was performed using Gaussian 09 distribution for Windows [53]. Geometry optimization and frequency calculations were completed with DFT method using the B3LYP functional. Geometry optimization and frequency calculations were done using the 6-311++G(2d,p) basis set for C, H, N, O, and S atoms, and LANL2DZ with ECP for Au atoms [4]. Calculated frequencies and intensities were scaled according to the procedure described elsewhere [4,54,55]. No imaginary wavenumbers were obtained in the calculated spectra.

### 4.4. Synthesis of MOPHE Compound

**Figure 7:**
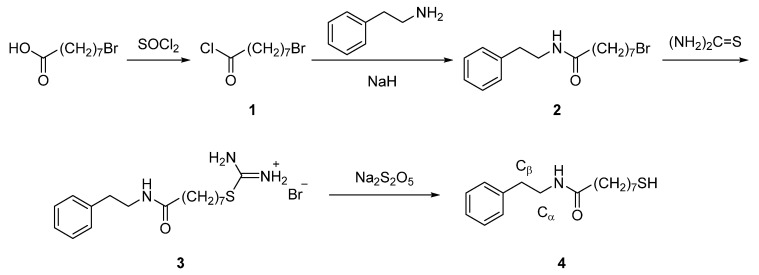
Synthesis route of 8-mercapto-*N*-(phenethyl)octanamide (**4**, MOPHE).

In this work, we synthesized bifunctional thiol molecule 8-mercapto-*N*-(phenethyl)octanamide (MOPHE) able to form self-assembled monolayers at the gold substrate. The synthesis route of this compound is shown in Figure 7.

*General procedures.* 8-Bromooctanoic acid (97%) and 2-phenylethan-1-amine (99%) were obtained from Sigma-Aldrich Chemie GmbH (Munich, Germany) and Across Organics (Geel, Belgium), respectively, and were used without additional purification. ^1^H and ^13^C NMR spectra were recorded in CDCl_3_ on Varian Unity Inova (300 MHz) spectrometer (Varian, Palo Alto, CA, USA) at a frequency of 299.75 MHz and 75.37 MHz for ^1^H and ^13^C, respectively; spectra were referenced using the solvent signal as internal standard (^1^H NMR: δ = 7.26 ppm; ^13^C NMR: δ = 77.0 ppm).

*8-Bromo-N-(phenethyl)octanamide* (**2**). Mixture of 8-bromooctanoic acid (1.1 g, 5 mmol), thionyl chloride (0.72 mL, 10 mmol) and dimethylformamide (0.2 mL) was stirred at room temperature for 15 h and evaporated. The obtained 8-bromooctanoylchloride (**1**) was used in the subsequent step without further purification. Solution of **1** in dry tetrahydrofuran (20 mL) was added to a mixture of 2-phenylethan-1-amine (0.6 g, 5 mmol) and NaH (0.3 g, 60% dispersion in mineral oil) in dry tetrahydrofuran (30 mL) at 0 °C. The reaction mixture was stirred at 0 °C for 2 h and then at room temperature for 20 h. The reaction mixture was then diluted with chloroform and washed with 0.5 M HCl. Organic phase was dried over anhydrous MgSO_4_, filtered and evaporated. Purification by crystallization from *n*-hexane afforded compound **2** (1 g, 61.3%). mp. 57–58 °C; ^1^H NMR (CDCl_3_): 1.26–1.36 (4H, m), 1.37–1.46 (2H, m), 1.59 (2H, p, *J* = 7.3 Hz), 1.84 (2H, p, *J* = 6.9 Hz), 2.12 (2H, t, *J* = 7.5 Hz), 2.82 (2H, t, *J* = 6.8 Hz), 3.40 (2H, t, *J* = 6.8 Hz), 3.53 (2H, q, *J* = 6.8 Hz), 7.1–7.33 (5H, m);. Found (%): C 58.67, H 7.62, Br 24.26. C_16_H_24_BrNO. Requires (%): C 58.90, H 7.41, Br 24.49.

*8-Mercapto-N-(phenethyl)octanamide (MOPHE)* (**4**). A solution of **2** (1 g, 3.1 mmol) and thiourea (0.3 g, 4 mmol) in acetonitrile (50 mL) was refluxed for 14 h, then cooled to room temperature and evaporated to dryness. The remaining residue was triturated with acetone and filtered. The obtained compound **3** was used in the subsequent step without further purification. To a solution of compound **3** (0.7 g, 1.7 mmol) in water (40 mL) and chloroform (40 mL), Na_2_S_2_O_5_ (0.4 g, 2 mmol) was added and the resulting mixture was refluxed under argon atmosphere for 7 h. Cooled reaction mixture was extracted with chloroform, combined organic phase was dried over anhydrous Na_2_SO_4_, filtered and evaporated. Purification by recrystallization from *n*-hexane afforded compound **4** (0.4 g, 41%). mp 58–60 °C; ^1^H NMR (CDCl_3_): 1.25–1.44 (7H, m), 1.54–1.64 (4H, m), 2.11 (2H, t, *J* = 7.5 Hz), 2.52 (2H, q, *J* = 7.5 Hz), 2.82 (2H, t, *J* = 6.8 Hz), 3.51 (2H, q, *J* = 6.8 Hz), 7.17–7.35 (5H, m); ^13^C NMR (CDCl_3_): 24.6, 25.6, 28.2, 28.8, 29.1, 33.9, 35.7, 36.8, 40.5, 126.5, 128.7, 128.8, 138.9, 172.9. Found (%): C 68.91, H 9.22, S 11.25. C_16_H_25_NOS. Requires (%): C 68.77, H 9.02, S 11.47. 

## 4.5. Synthesis of MOPHE-D_5_ Compound

**Figure 8:**
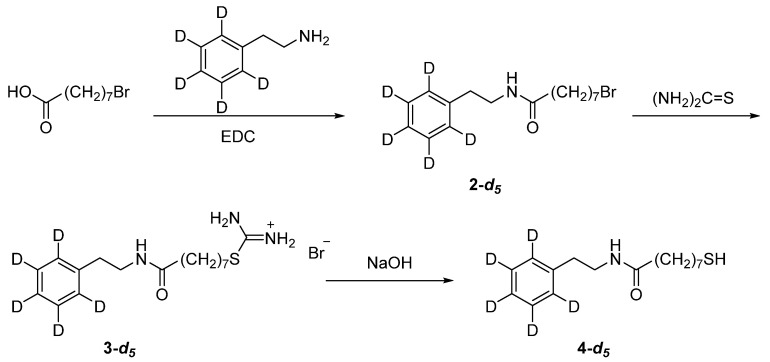
Synthesis route of 8-mercapto-*N*-(phenethyl)octanamide-*d*_5_ (**4-*d*_5_**, MOPHE-D_5_).

To afford reliable assignments of vibrational bands, we synthesized the Phe ring deuterated analog of the studied compound, MOPHE-D_5_. Synthesis route of this compound is shown in Figure 8.

*General Procedures.* Bromobenzene-*d*_5_ (99.5 atom%), *N*-(3-dimethylaminopropyl)-*N*’-ethylcarbodiimide hydrochloride (EDC) and 8-bromooctanoic acid were purchased from Sigma-Aldrich Chemie GmbH, Apollo Scientific (Stockport, UK) and Fluorochem (Hadfield, UK), respectively. 2-Phenylethan-1-amine-*d*_5_ was prepared from bromobenzene-*d*_5_ by following the reported procedures [56]. Dichloromethane and trimethylamine were dried and distilled from calcium hydride under argon before use. Thin layer chromatography was carried out on Kieselgel 60 F_254_ (Merck, Darmstadt, Germany) sheets coated with silica gel, and ZEOprep 60 silica gel (35–70 μm) (Apollo Scientific) was used for column chromatography.

^1^H and ^13^C NMR spectra were recorded in CDCl_3_ on Avance III (400 MHz) spectrometer (Bruker BioSpin GmbH, Rheinstetten, Germany) operating at a frequency of 400.13 and 100.61 MHz for ^1^H and ^13^C, respectively; spectra were referenced using the solvent signal as internal standard (^1^H NMR: δ = 7.26 ppm; ^13^C NMR: δ = 77.0 ppm). GC-MS analyses were performed with a Shimadzu GCMS-QP2010Ultra Plus (Kyoto, Japan), chromatographic separation was achieved on a Rxi^®^-5 ms column (30 m × 0.25 mm I.D., 0.25 μm film thickness (Restek, Bellefonte, PA, USA) using helium as carrier gas at 40 cm/s in a constant linear velocity mode. Temperature program: 50 °C (1 min), from 50 °C to 320 °C (25 °C/min), from 320 to 330 °C (50 °C/min), 330 °C (3.7 min). The temperatures of injector, interface, and ion source were 250, 330, and 250 °C, respectively. Detection was operated by selected ion monitoring (SIM) mode (EI mode), data were collected and analyzed using the GC-MS solution version 2.71 (Shimadzu, Kyoto, Japan).

*8-Bromo-N-(phenethyl)octanamide-d_5_* (**2-*d*_5_**). Triethylamine (2.1 mL, 15 mmol) and *N*-(3-dimethylaminopropyl)-*N*’-ethylcarbodiimide hydrochloride (EDC; 2.3 g, 12 mmol) were added to a solution of 2-phenylethan-1-amine-*d*_5_ (1.26 g, 10 mmol) and 8-bromooctanoic acid (2.68 g, 12 mmol) in dry dichloromethane (100 mL) at 0 °C. The reaction mixture was stirred at 0 °C for 1 h and then at room temperature for 20 h. The reaction mixture was then diluted with dichloromethane (100 mL) and washed successively with 0.5 M HCl (2 × 100 mL), water (100 mL), saturated NaHCO_3_ (100 mL), and water (100 mL). The organic phase was dried over anhydrous Na_2_SO_4_, filtered and evaporated. Purification of the obtained residue by column chromatography on silica gel with a mixture of hexane-ethyl acetate (6:4, R_f_ = 0.24) afforded **2-*d*_5_** (2.51 g, 76%) as a clear oil, which solidifies on standing. Yellowish solid, mp. 57–59 °C; ^1^H NMR (CDCl_3_): 1.25–1.45 (6H, m), 1.59 (2H, p, *J* = 7.5 Hz), 1.84 (2H, p, *J* = 6.8 Hz), 2.11 (2H, t, *J* = 7.5 Hz), 2.82 (2H, t, *J* = 6.8 Hz), 3.39 (2H, t, *J* = 6.8 Hz), 3.52 (2H, q, *J* = 6.8 Hz), 5.47 (1H, br s); ^13^C NMR (CDCl_3_): 25.5, 27,9, 29.0, 32.7, 33.9, 35.6, 36.7, 40.4, 138.7, 172.9; GC-MS: t_R_ = 11.475 min (*m*/*z* 330/332 (2.4/2.3%), [M]^+●^).

*8-Mercapto-N-(phenethyl)octanamide-d_5_* (**4-*d*_5_**). A solution of **2-*d*_5_** (2.45 g, 7.4 mmol) and thiourea (620 mg, 8.14 mmol) in ethanol (24 mL) was refluxed overnight under argon atmosphere, then cooled to room temperature and evaporated to dryness. The obtained crude **3-*d*_5_** was used in the subsequent step without further purification. Crude **3-*d*_5_** was suspended in degassed aqueous NaOH solution (326 mg, 8.14 mmol in 24 mL water) and the resulting mixture was stirred at 100 °C under argon atmosphere for 2 h. Cooled reaction mixture was extracted with dichloromethane (3 × 20 mL), combined organic phase was dried over anhydrous Na_2_SO_4_, filtered and evaporated. Purification of the obtained residue by column chromatography on silica gel with a mixture of dichloromethane-methanol (99:1, R_f_ = 0.18) afforded **4-*d*_5_** (1.54 g, 73%) as a clear oil, which solidifies on standing. Colorless solid, mp. 56–57 °C; ^1^H NMR (CDCl_3_): 5.45 (1H, br s), 3.52 (2H, q, *J* = 6.8 Hz), 2.81 (2H, t, *J* = 6.8 Hz), 2.51 (2H, q, *J* = 7.4 Hz), 2.11 (2H, t, *J* = 7.5 Hz), 1.62–1.55 (4H, m), 1.40–1.25 (6H, m), 1.32 (1H, t, *J* = 7.7. Hz); ^13^C NMR (CDCl_3_): 24.6, 25.6, 28.1, 28.7, 29.1, 33.9, 35.6, 36.7, 40.4, 138.7, 172.9; GC-MS: t_R_ = 11.412 min (*m*/*z* 251 (54%), [M-SH]^+●^).

## Figures and Tables

**Table 1:** Frequencies (cm^−1^) and assignments of main infrared absorption bands of MOPHE (8-mercapto-*N*-(phenethyl)octanamide) and MOPHE-D_5_ (8-mercapto-*N*-(phenethyl)octanamide-*d*_5_).

Solid State		Calculated		Assignments
MOPHE	MOPHE-D_5_	MOPHE	MOPHE-D_5_	
3309 vs	3309 vs	3568	3568	ν(N−H) Amide-A
3027 w	2275 w	3124	3124	ν(=C–H) Phe
2926 s	2926	3004	3004	ν_as_(CH_2_) Chain
2852 s	2852 s	2973	2973	ν_s_(CH_2_) Chain
2558 w	2258 v	2635	2635	ν(S−H) Thiol
1640 vs	1640 vs	1713	1713	ν(C=O) + δ(NH) Amide-I
1546 vs	1546 vs	1525	1525	ν(C−N) + δ(NH) Amide-II
1496 m	1383 m	1481	1403	ν(C=C) + β(CH) Phe (F19a) ^a^
1465 m	1465 m	1466	1466	δ(CH_2_) Chain (scissoring)
1454 w	1383 w	−	−	ν(C=C) + β(CH) Phe (F19b)
1418 w	1418 w	1392	1392	δ(CH_2_) Chain (scissoring)
1328 m	1328 m	1354	1354	δ(CH_2_) Chain
1257 w	1257 w	1262	1262	δ(CH_2_) Chain
1249 m	1249 m	1241	1241	ν(C−N) + δ(CNH) Amide-III
1195 m	1195 m	1206	1207	δ(CH_2_) Chain
1030 w	821 w	1048	837	β(CH) Phe (F18a)
749 s	546 s	763	554	γ(CH) Phe (F11)
707 br,m	707 br, m	−	−	γ(NH) Amide V
699 s	447 s	714	452	γ(CH) Phe (F4)

Abbreviations: ν, stretching; δ, deformation; β, in-plane bending; γ, out-of-plane bending; vs, very strong; s, strong; m, middle; w, weak; br, broad; s, symmetric; as, asymmetric. ^a^ The Wilson notation is used for the description the characteristic vibrational modes of the Phe ring.
